# Emerging Roles of Pleckstrin-2 Beyond Cell Spreading

**DOI:** 10.3389/fcell.2021.768238

**Published:** 2021-11-17

**Authors:** Gengchen Wang, Qian Zhou, Yan Xu, Baobing Zhao

**Affiliations:** ^1^Department of Pharmacology, School of Pharmaceutical Sciences, Cheeloo College of Medicine, Shandong University, Jinan, China; ^2^Key Laboratory of Chemical Biology (Ministry of Education), School of Pharmaceutical Sciences, Cheeloo College of Medicine, Shandong University, Jinan, China

**Keywords:** pleckstrin-2, cell spreading, erythropoiesis, metastasis, tumorigenesis

## Abstract

Pleckstrin-2 is a member of pleckstrin family with well-defined structural features that was first identified in 1999. Over the past 20 years, our understanding of PLEK2 biology has been limited to cell spreading. Recently, increasing evidences support that PLEK2 plays important roles in other cellular events beyond cell spreading, such as erythropoiesis, tumorigenesis and metastasis. It serves as a potential diagnostic and prognostic biomarker as well as an attractive target for the treatment of cancers. Herein, we summary the protein structure and molecular interactions of pleckstrin-2, with an emphasis on its regulatory roles in tumorigenesis.

## Introduction

**FIGURE 1:**
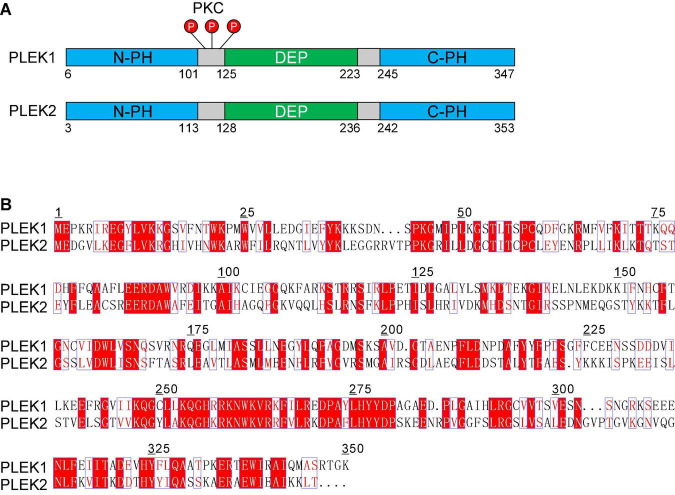
The pleckstrin family of proteins. **(A)** Structural domain overview of human PLEK1 and PLEK 2. Both PLEK1 and PLEK1 contain two pleckstrin homology (PH) domains (PH, shown in blue) at the amino- and carboxyl-terminal and a central disheveled-Egl-10-pleckstrindomain (DEP, shown in green). There are three sites (Ser113, Thr114, and Ser117) between DEP domain and the N-terminal PH domain of PLEK1, which can be phosphorylated by PKC (shown in red) **(B)** Comparison of the deduced amino acid sequence of human PLEK1 and PLEK2. The similar amino acids are shown in red, and the same ones are highlighted as red.

Pleckstrin was first initially described as a prominent substrate of protein kinase C (PKC) in hematopoietic cells. In its naming, “PLEC” is derived from platelet and leukocyte C kinase substrate, and “KSTR” is derived from the amino acid sequence KSTR. Pleckstrin protein sequences are highly conserved across human and mouse, which is about 97% homologous to each other. Accordingly, the protein structures and functions of these two different species are also almost same. Pleckstrin-1 (PLEK1), specifically expressed in hematopoietic cells, is composed of two pleckstrin homology (PH) domains at the amino- and carboxyl-terminal and a central disheveled-Egl-10-pleckstrin (DEP) domain (Figure 1A). As a major substrate of PKC in platelets and leukocytes, the phosphorylation of PLEK1 was used as a marker for platelet activation (Hu et al., 1999; Inazu et al., 1999).

Pleckstrin-2 (PLEK2) is another member of pleckstrin family, which is 65% homologous and 39% identical to paralog PLEK1 (Figure 1B). Similar to PLEK1, PLEK2 harbors a central DEP domain flanked by two PH domains (Figure 1A). However, PLEK2 is widely expressed, especially in thymus, stomach, large and small bowels, and prostate (Hu et al., 1999; Inazu et al., 1999).

Both isoforms can induce the formation of large lamellipodia and peripheral ruffle of cells, thereby facilitating cell spreading, in which PLEK1 is entirely regulated by its phosphorylation by PKC (Ma and Abrams, 1999; Roll et al., 2000). In contrast, PLEK2 is not efficiently phosphorylated by PKC but rather by the local generation of PI3K-phosphorylated phospholipids (Bach et al., 2007; Hamaguchi et al., 2007). Recently, roles of PLEK2 in other cellular events beyond cell spreading are gradually being identified. Several lines of evidences suggest that PLEK2 is a potential therapeutic target for the treatment of cancers (Zhao et al., 2018; Shen et al., 2019; Wu et al., 2020; Liu et al., 2021; Wang et al., 2021a; Yang et al., 2021).

## Structure of PLEK2

Three-dimensional (3D) protein structures have contributed enormously to our understanding of atomic and molecular structure, and help delineate complex macromolecules and their functions, as well as how they operate in the real world. Although the amino sequence of PLEK2 has been characterized with well-defined domains, its protein architecture is poorly investigated. Only the solution structures of C-terminal PH domain and DEP domain of PLEK2 had been reported, respectively (Table 1). Due to the similarity in the structure of the two proteins, the studies on structure of PLEK1 may provide insights for the study of PLEK2. C-terminal PH domain of human PLEK1 (PDB code:1XX0, 1 × 05) was first reported by two independent groups in 2005 (Edlich et al., 2005). Its crystal structure was identified in 2006 (PDB code:1ZM0) (Jackson et al., 2006) and further confirmed by the crystal structure in complex with D-myo-Ins(1,2,3,5,6)P5 (PDB code:2I5F) and D-myo-Ins(1,2,3,4,5)P5 (PDB code:2I5C), respectively, in 2007 (Jackson et al., 2007). Furthermore, it has been suggested that myo-inositol pentakisphosphates regulate the interaction between PH domain and phosphoinositides through direct competition binding to PH domain (Jackson et al., 2007). Although the solution structures of DEP and N-PH domains had been published, their physical structures need to be further confirmed by protein crystal.

**TABLE 1:** Three-dimensional protein structures of PLEK2 and PLEK1.

**Protein**	**Domain**	**Organisms**	**Type**	**Method**	**PDB ID**
PLEK2	C-PH	Homo	Solution	NMR	1X1G
	DEP	Mus	Solution	NMR	1V3F
PLEK1	C-PH	Homo	Solution	NMR	1XX0/1X05
	C-PH	Homo	Crystal	X-RAY	1ZM0
	C-PH (IP5)	Homo	Crystal	X-RAY	2I5C/2I5F
	DEP	Homo	Solution	NMR	1W4M/2CSO
	DEP	Mus	Solution	NMR	1UHW
	N-PH	Homo	Solution	NMR	1LPS

The structures of PH domain and DEP domain had been well characterized, respectively, however, full-length structures of PLEK1 and PLEK2 are still largely unknown. Improvements in protein production, crystallization, as well as structure solution and refinement methods have brought the field to the verge of rapid protein structure determination. The major bottle neck to this process remains protein production and crystallization. It has been suggested that DEP interacts intramolecularly with the N-terminal PH domain to mediate membrane anchoring of PLEK1 (Civera et al., 2005). Such interaction may also occur between two PLEK molecules, which accounts for the formation of irregular polymers of PLEK2. This is further supported by the endogenous oligomerization of PLEK2 (Hamaguchi et al., 2007). Indeed, our group previously tried to acquire the protein crystal of PLEK2 but with no success. The failure was due to the difficulty to purify the protein monomer from irregular polymers of PLEK2.

Examining the crude 3D model built with the sequence for mouse PLEK2, two putative small molecule binding grooves appear to exist near K13 and R14 residues (N-PH domain) and at K256 residues (C-PH domain), respectively. Consistently, these sites have been suggested to be response for the phospholipid binding domains (Bach et al., 2007; Jackson et al., 2007). Furthermore, at N156,N157 and D166 (DEP domain) also a potential ligand binding groove exists in the preliminary model.

## Biological Function of PLEK2

**FIGURE 2:**
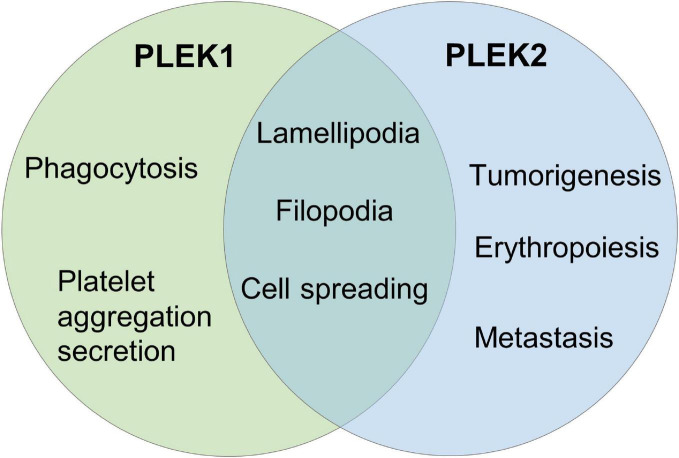
The biological function of PLEK2 and PLEK1.

Due to the highly similarity of protein structural features, it is not surprise that both of these two pleckstrin proteins regulate cell protrusions such as lamellipodia and filopodia, which are important for cellular shape change and spreading (Figure 2). Meanwhile, PLEK2 exhibits distinct roles in other cellular events through the individual molecular interactions and regulatory mechanisms (Table 2).

**TABLE 2:** The biological functions and interacting molecules of PLEKs.

**Protein**	**Function**	**Interaction partners**	**Method**	**References**
PLEK2	Cell spreading	Actin	OE	Hu et al., 1999
		PI(3,4)P2/PI(3,4,5)P3	KD/OE	Dowler et al., 2000; Hamaguchi et al., 2007
	Inflammation	PI(3,4)P2/PI(3,4)P3	OE	Bach et al., 2007
		F-actin	OE	Bach et al., 2007
	Erythropoiesis	Actin/Cofilin	OE/KD	Zhao et al., 2014
		Rac GTPase	KD/KO mice	Feola et al., 2021
	Tumorigenesis	SHIP2	OE/KD	Wu et al., 2020
		EGFR	OE/KD	Shen et al., 2019
		JAK2/STAT5	KD/KO mice	Zhao et al., 2018
PLEK1	Cell spreading	Rac GTPase	OE	Ma and Abrams, 1999
		Integrin	OE	Roll et al., 2000
	Platelet activation	NA	OE	Baig et al., 2009a, b; Lian et al., 2009
	Phagocytosis	NA	OE	Brumell et al., 1999
	Inflammation	NA	OE	Brumell et al., 1997; Kienzle et al., 1997; Ding et al., 2007

*OE, Overexpression; KD, Knockdown; KO, Knockout; NA, not applicable.*

### Pleckstrin Homology Domain and Disheveled-Egl-10-Pleckstrin Domain of PLEK2

PLEKs are best known for containing two PH domains at the amino- and carboxyl-terminal and a central DEP domain. PH domain is a functional domain consisting of approximately 120 amino acids, which has been found in a variety of proteins involved in cellular signaling or cytoskeletal functions. It plays an important role in cellular signal transduction by recruiting host proteins to cell membrane via phospholipid binding (Lemmon et al., 1996). DEP, a globular protein domain that is present in about ten human protein families, has also been shown to facilitate the translocation of the host proteins to the plasma membrane through diverse mechanisms. In addition, DEP domain is also involved in the interaction of host proteins with various partners at the membrane including phospholipids and membrane receptors (Consonni et al., 2014). Given that neither motif has enzymatic activity, the function of PLEKs most probably involves multiple intermolecular interactions mediated by these domains.

As a prominent substrate of PKC, the biological function of PLEK1 largely depends on its phosphorylation. There are three sites (Ser113, Thr114, and Ser117) between DEP domain and the N-terminal PH domain, which can be phosphorylated by PKC (Figure 1A; Abrams et al., 1995). N-terminal PH domain binds to certain phospholipids and then regulates PLEK1 anchoring to membrane (Ma et al., 1997). Furthermore, DEP domain of PLEK1 interacts with its N-terminal PH domain intramolecularly, participating in membrane localization of PLEK1 indirectly, after the phosphorylation by PKC (Civera et al., 2005).

Unlike PLEK1, PLEK2 is a poor substrate of PKC and regulated by the local generation of phosphatidylinositol 3-kinase (PI3K)-phosphorylated phospholipids (Dowler et al., 2000; Bach et al., 2007). Both PH domains contribute to lamellipodia formation and membrane anchoring of PLEK2 via the phospholipid binding (Hu et al., 1999; Bach et al., 2007). Importantly, PLEK2 colocalizes with actin to induce the reorganization of cytoskeleton and peripheral ruffle formation via its PH domains. Although DEP domain is not essential for the phospholipid binding of PLEK2, it plays a role in membrane ruffles and cell spreading by cooperating with PH domains (Bach et al., 2007).

### PLEK2 in Cell Spreading

PLEKs was firstly found to be involved in cytoskeletal rearrangement. Expression of PLEK1 results in cell spreading and rearrangement of cytoskeleton characterized by morphologic change such as formation of peripheral membrane ruffles or dorsal projection, which is dependent upon the Rac activity and integrin binding (Ma and Abrams, 1999; Roll et al., 2000; Bach et al., 2007). PLEK2 expressed in a variety of adherent cells is concentrated at the cell membrane, including the membrane of lamellipodia, ruffles and other membrane protrusions (Hu et al., 1999; Hamaguchi et al., 2007). Although Rac is also suggested to co-precipitate with PLEK2, actin rearrangements induced by PLEK2 is dependent of PI3K but not the interaction of Rac and PLEK2 (Hamaguchi et al., 2007). PLEK2 interacts with membrane-associated phosphatidylinositols generated by PI3K, to participate in actin rearrangement and coordinate with actin cytoskeleton, which causes cell spreading (Bach et al., 2007; Hamaguchi et al., 2007). Accordingly, a PLEK2 mutant incapable of binding to the PI 3-kinase products did not show any effect on actin rearrangement (Hamaguchi et al., 2007).

### PLEK2 in Inflammation

It has been well documented that rearrangement of cytoskeleton is fundamental to the immune synapse formation in lymphocytes (Dustin and Cooper, 2000). PLEK2 functions as a regulatory node for PI3K-mediated cytoskeletal reorganization in lymphocyte spreading and immune synapse formation (Bach et al., 2007). PLEK2 is recruited to the cell membrane and induces matrix-dependent lamellipodia formation and cell spreading upon stimulation of the T-cell receptor or α4β1, which is dependent upon the binding of PI3K-generated membrane-bound phospholipids. On the other hand, PLEK2 colocalizes with F-actin and organizes the cytoskeleton to promote lymphocyte spreading and immune synapse formation.

Given that PLEK1 is exclusively expressed in monocytes, macrophages, lymphocytes, and granulocytes, it is acceptable for the roles of PLEK1 in inflammation. In addition to the critical effects on platelet aggregation and secretion that was mediated by PKC, PI3K and Actin assembly (Baig et al., 2009a, b; Lian et al., 2009). PLEK1 has shown to be highly phosphorylated and accumulated on the cell membrane to regulate phagocytosis of macrophages in response to bacterial LPS and IFNγ, however, the association of PLEK1 with phagosomes is independent of its phosphorylation (Brumell et al., 1999). Similar phosphorylation and subcellular redistribution of PLEK1 are also occurred in the stimulation of neutrophils and B lymphocytes (Brumell et al., 1997; Kienzle et al., 1997). Moreover, PLEK1 functions as a critical molecule in modulating proinflammatory cytokine secretion by mononuclear phagocytes of diabetics (Ding et al., 2007). Consistently, it has also been suggested that PLEK1 is involved in the development and progression periodontitis and other chronic inflammatory diseases (Lundmark et al., 2015; Song et al., 2015).

### PLEK2 in Erythropoiesis

PLEK2 has shown high expression level in erythroid cells and a critical role in erythropoiesis that starts with erythroid progenitors committed from hematopoietic stem cells to mature red blood cells (Zhao et al., 2014). Binding of PLEK2 prevents cofilin’s mitochondrial entry and consequent apoptosis of early stage erythroblasts in response to the high level of reactive oxygen species (ROS) (Zhao et al., 2016). On the other hand, PLEK2 serves as a docking site and regulates actin cytoskeleton integrity that is critical for erythroblasts proliferation and differentiation, and terminal enucleation (Zhao et al., 2014). However, PLEK2-knockout mice were largely normal at the young age but showed mild anemia with age due to ineffective erythropoiesis (Zhao et al., 2018), suggesting that PLEK2 plays a critical role in stressed erythropoiesis. Accordingly, loss of PLEK2 led to the embryonic lethality due to the worsening ineffective erythropoiesis in β-thalassemic mice (Feola et al., 2021), and also reverted the excessive erythropoiesis in the polycythemia vera mouse model (Zhao et al., 2018).

During terminal erythropoiesis, the nucleus is gradually condensed, and the highly condensed nucleus in the orthochromatic erythroblast is extruded out from the erythroblast (Ji, 2015). The erythroid membrane and cytoskeleton also undergo remodeling and dynamics dynamic that is critical for terminal enucleation (Mei et al., 2020). PLEK2 is not required for cell differentiation in the late stage of terminal erythropoiesis but still critical for enucleation (Zhao et al., 2014). It is demonstrated that PLEK2 regulated actin dynamics through interaction with cofilin. Given that PLEK2 showed colocalization with Rac (Hamaguchi et al., 2007), it is also possible that PLEK2 functions through Rac GTPases to regulate enucleation. Rac GTPases has been not only shown to regulate actin rearrangement, but also reported to regulate the formation of contractile actin ring between the pyknotic nucleus and incipient reticulocyte through formin protein mDia2 (Ji et al., 2008, 2011).

### PLEK2 in Tumorigenesis

#### The Role of PLEK2 in Hematological Malignancy

Myeloproliferative neoplasms (MPNs) are a group of bone marrow diseases with excessive production of blood cells, increasing risk of arterial or venous thrombosis, and a propensity to transform into acute myeloid leukemia. JAK2^*V*617*F*^ mutation is the leading cause of the Philadelphia-chromosome-negative MPNs. Recently, PLEK2 has been identified to be a downstream effector for JAK2/STAT5 signaling in hematopoietic cells upon the stimulation of corresponding cytokines (Zhao et al., 2018). Importantly, it is upregulated in a JAK2^*V*617*F*^-positive MPN mouse model and in patients with MPNs. Loss of PLEK2 substantially ameliorates the myeloproliferative phenotypes, including erythrocytosis, neutrophilia, thrombocytosis and splenomegaly. Meanwhile, loss of PLEK2 reverts the JAK2^*V*617*F*^-induced widespread vascular occlusions and lethality, mainly through the reduction of whole-body red blood cell mass. Altogether, PLEK2 functions a key factor in the pathogenesis of JAK2^*V*617*F*^-induced MPNs, pointing to PLEK2 as a viable target for the treatment of MPNs.

In addition, a recent bioinformatics study has also suggested that PLEK2 is significantly associated with survival of patients, as well as a novel therapeutic target for multiple myeloma (Yang et al., 2020).

#### The Role of PLEK2 in Tumorigenesis and Metastasis

Increasing evidences suggest that PLEK2 plays a cancer-promoting role in tumorigenesis and metastasis (Table 3). PLEK2 expression has been demonstrated to be highly upregulated in several malignancies, and its knockdown leads to the inhibition of cancer cell proliferation, migration and invasion, including non-small cell lung cancer (Wu et al., 2020), gallbladder cancer (Shen et al., 2019), osteosarcoma (Liu et al., 2021), pancreatic cancer (Yang et al., 2021), gastric cancer (Wang et al., 2021a), and esophageal squamous cell carcinoma (Wang et al., 2021). Although two recent studies showed that PLEK2 mRNA expression is down-regulated in multiple myeloma bone marrow progenitor cells and prostate cancer, its expression is thought to be positively correlated with the poor prognosis (Wang et al., 2020; Yang et al., 2020). Furthermore, it has also been indicated to be associated with poor prognosis in other tumors. PLEK2 is identified as an prognosis factor for prostate cancer, lung adenocarcinoma and head and neck squamous cell carcinoma in independent bioinformatics analysis with gene signature-based risk assessment models (Yin et al., 2016; Jiang et al., 2020; Wang et al., 2020, 2021b; Zhang et al., 2020). Its expression in pancreatic cancer is positively correlated with the expression of insulin-like growth factor 2 mRNA binding protein IMP2 that is oncogenic protein known to be overexpressed in different types of cancers (Dahlem et al., 2019). A genome-wide expression profiling study suggested that PLEK2 expression is correlated with the metastasis of breast cancer (Naume et al., 2007) and melanoma (Luo et al., 2011). Moreover, PLEK2 has been reported to be highly correlated with long no coding RNA LOC541471 that serves a core role in the tumorigenesis of glioblastoma multiforme (Chen et al., 2019).

**TABLE 3:** Dysregulation of PLEK2 in cancer.

**Cancer**	**Expression**	**Level**	**Method**	**Prognosis**	**References**
MPN	mRNA/Protein	Up	QPCR/WB/IHC	NA	Zhao et al., 2018
Multiple myeloma	mRNA	Down	Bioinfor	+	Yang et al., 2020
Melanoma	mRNA	Up	Bioinfor	NA	Luo et al., 2011
Lung cancer	mRNA/Protein	Up	QPCR/WB/IHC	+	Wu et al., 2020
Gallbladder cancer	mRNA/Protein	Up	QPCR/WB/IHC	+	Shen et al., 2019
Breast carcinoma	mRNA	Up	Bioinfor	NA	Guillaud-Bataille et al., 2009
Glioblastoma multiforme	mRNA	Up	Bioinfor	NA	Chen et al., 2019
Lung adenocarcinoma	mRNA	Up	Bioinfor	NA	Jiang et al., 2020; Zhang et al., 2020
Lung cancer	mRNA	Up	Bioinfor	+	Yin et al., 2016
Pancreatic cancer	mRNA/Protein	Up	QPCR/WB/IHC	NA	Yang et al., 2021
Pancreatic Cancer	mRNA	Up	Bioinfor	NA	Dahlem et al., 2019
Osteosarcoma	mRNA/Protein	Up	QPCR/WB/IHC	NA	Liu et al., 2021
Breast cancer	mRNA	Up	Bioinfor	NA	Naume et al., 2007
Gastric cancer	mRNA/Protein	Up	QPCR/WB/IHC	+	Wang et al., 2021a
Prostate cancer	mRNA	Down	Bioinfor	-	Wang et al., 2020
Oesophageal squamous cell carcinoma	mRNA/Protein	Up	QPCR/WB/IHC	+	Wang et al., 2021
Head and neck squamous cell carcinoma	mRNA/Protein	Up	WB/Bioinfor	+	Wang et al., 2021b

*Bioinfor, bioinformatics analysis; NA, not applicable.*

The epithelial-to-mesenchymal transition (EMT) process is a crucial mechanism in the progression of tumor metastasis (De Craene and Berx, 2013; Dongre and Weinberg, 2019). Regulation of EMT is implicated in the tumor cell invasion and migration during metastasis. During EMT, cells progressively redistribute or downregulate their apical and basolateral epithelial-specific proteins, such as E-cadherin, catenin, and cytokeratin, and re-express mesenchymal molecules, such as vimentin, fibronectin, and N-cadherin. These changes lead to the abrogation of cell-cell junctions and the gain of cell invasive and migratory capabilities, which involve a dramatic reorganization of the actin cytoskeleton and the concomitant formation of membrane protrusions including lamellipodia, filopodia and invadopodium and podosomes (Yilmaz and Christofori, 2009). PLEK2 expression has been reported to be positively correlated with migration and invasion in numerous tumors (Yin et al., 2016; Shen et al., 2019; Wu et al., 2020; Zhang et al., 2020). Given that PLEK2 exerts strong regulatory effects on actin cytoskeletal actin rearrangement and subsequent formation of large lamellipodia and the peripheral ruffle of cells, PLEK2 upregulation may directly lead to enhanced invasive capability of cancer cells.

Despite conclusive evidence supporting PLEK2 is involved in cell migration and invasion, its regulatory roles in tumor cell invasion and metastasis remains largely limited. It has been demonstrated that PLEK2 interacts with epidermal growth factor receptor (EGFR) and suppresses EGFR ubiquitination mediated by c-CBL, leading to downstream CCL2 transcriptional overexpression and EMT process activation in gallbladder cancer (Shen et al., 2019). Additionally, PLEK2 expression is upregulated by TGF−β stimulation through ELK1 and Smad2/3 in non-small cell lung cancer and esophageal squamous cell carcinoma, respectively (Wu et al., 2020; Wang et al., 2021). It interacts with and targets SHIP2 for degradation that activates PI3K/AKT signaling to mediate lung cancer cell migration and vascular invasion. Similarly, PI3K/AKT signaling mediated by PLEK2 is also involved in osteosarcoma tumorigenesis (Liu et al., 2021) and metastasis and in self-renewal and proliferation of pancreatic cancer stem cells (Yang et al., 2021).

## Molecular Interaction Partners of PLEK2

Interaction partners are essential for the study of molecular mechanism of proteins in cell biology. Our understanding of PLEK2 function is limited mainly due to the poorly identification of interaction partners (Table 2). As mentioned above, PLEK2 interacts with PI (3,4,5) P3 and PI (3,4) P2 generated by PI3K, to anchor on the cell membrane and regulate lamellipodia formation and cell spreading (Bach et al., 2007; Hamaguchi et al., 2007). On the other hand, PLEK2 directly binds to Actin (Hu et al., 1999; Zhao et al., 2014), or interacts with Rac GTPase (Hamaguchi et al., 2007; Feola et al., 2021), to regulate the cytoskeleton dynamics. Similarly, Rac has also been reported to be interplayed with PLEK1 to reorganize the cytoskeleton in addition to integrin (Ma and Abrams, 1999; Roll et al., 2000).

SHIP2, well known to negatively regulate the signaling via dephosphorylation of the 3-position of PI(3,4,5)P3 to generate PI(4,5)P2, is one of the two recently identified interaction partners of PLEK2. PLEK2 mediates degradation of SHIP2 in a ubiquitin-dependent manner, which is further activated PI3K/AKT signaling to promote lung cancer metastasis and vascular invasion (Wu et al., 2020). The other one is EGFR, which is involved in the EMT process activation in gallbladder cancer. PLEK2 interacts with EGFR to protect it from proteasomal mediated degradation, leading to constitutive activation of EGFR signaling (Shen et al., 2019).

## Concluding Remarks

**FIGURE 3:**
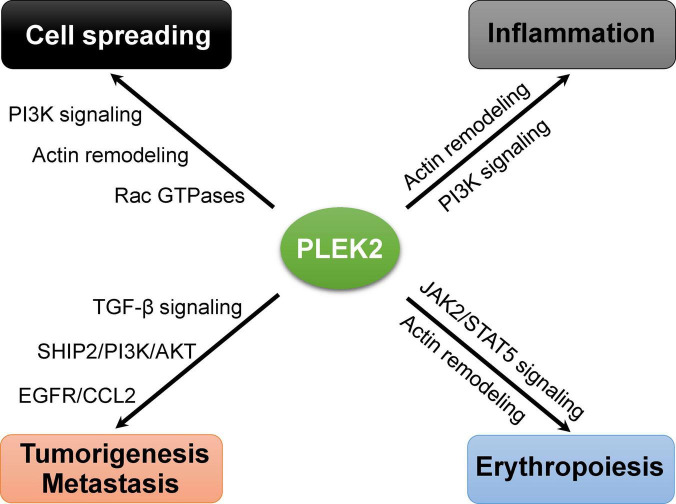
Emerging roles of PLEK2 in the regulation of different biological events.

As our understanding of the biological functions of PLEK2 increases, it is becoming clear that PLEK2 plays important roles in other cellular events beyond cell spreading, such as inflammation, erythropoiesis, tumorigenesis and metastasis (Figure 3). However, its regulatory roles in these cellular activities remains poorly understood. Further identification of interaction partners and exact regulatory mechanisms that are involved in such cellular process regulation, is required for a full understanding of the functional roles of PLEK2 and the development of targeted therapy.

The role of PLEK2 in tumor development is gradually being recognized, and it serves as a potential diagnostic and prognostic biomarker as well as an attractive target for the treatment of cancers. However, there is no reported small molecule inhibitor for PLEK1 or PLEK2. Therefore, the development of small molecules that modulate the function of PLEK2 is not only beneficial for the treatment of diverse cancers, but also provides extremely useful tools for studying PLEK2 functions as a powerful complementary method to the genetic methods.

Importantly, the three-dimensional protein structure is essential for designing and generating small molecules targeting PLEK2. Although there is no reported full-length structure of PLEK2, the prediction of the 3D structure of PLEK2 based on the solution structures of fragments through homology modeling methods (Vyas et al., 2012; Jaramillo-Martinez et al., 2021), could provide potential small molecule binding pockets for the high-throughput virtual screening and subsequent drug discovery. This promises to enable to validate and use PLEK2 as drug targets in the future.

## Author Contributions

GW, QZ, and BZ conceived the manuscript. GW and BZ wrote the manuscript. GW and YX contributed to crafting figures. GW, YX, and BZ reviewed and edited the manuscript. All authors listed have made a substantial, direct and intellectual contribution to work, and approved it for publication.

## Conflict of Interest

The authors declare that the research was conducted in the absence of any commercial or financial relationships that could be construed as a potential conflict of interest.

## Publisher’s Note

All claims expressed in this article are solely those of the authors and do not necessarily represent those of their affiliated organizations, or those of the publisher, the editors and the reviewers. Any product that may be evaluated in this article, or claim that may be made by its manufacturer, is not guaranteed or endorsed by the publisher.

## References

[B1] Abrams C. S. Zhao W. Belmonte E. Brass L. F. (1995). Protein kinase C regulates pleckstrin by phosphorylation of sites adjacent to the N-terminal pleckstrin homology domain. *J. Biol. Chem.* 270 23317–23321. 10.1074/jbc.270.40.23317 7559487

[B2] Bach T. L. Kerr W. T. Wang Y. Bauman E. M. Kine P. Whiteman E. L. (2007). PI3K regulates pleckstrin-2 in T-cell cytoskeletal reorganization. *Blood* 109 1147–1155. 10.1182/blood-2006-02-001339 17008542 PMC1785144

[B3] Baig A. Bao X. Haslam R. J. (2009a). Proteomic identification of pleckstrin-associated proteins in platelets: possible interactions with actin. *Proteomics* 9 4254–4258. 10.1002/pmic.200900060 19722192

[B4] Baig A. Bao X. Wolf M. Haslam R. J. (2009b). The platelet protein kinase C substrate pleckstrin binds directly to SDPR protein. *Platelets* 20 446–457. 10.3109/09537100903137314 19852682

[B5] Brumell J. H. Craig K. L. Ferguson D. Tyers M. Grinstein S. (1997). Phosphorylation and subcellular redistribution of pleckstrin in human neutrophils. *J. Immunol.* 158 4862–4871. 9144502

[B6] Brumell J. H. Howard J. C. Craig K. Grinstein S. Schreiber A. D. Tyers M. (1999). Expression of the protein kinase C substrate pleckstrin in macrophages: association with phagosomal membranes. *J. Immunol.* 163 3388–3395. 10477609

[B7] Chen X. Pan C. Xu C. Sun Y. Geng Y. Kong L. (2019). Identification of survivalassociated key genes and long noncoding RNAs in glioblastoma multiforme by weighted gene coexpression network analysis. *Int. J. Mol. Med.* 43 1709–1722. 10.3892/ijmm.2019.4101 30816427 PMC6414176

[B8] Civera C. Simon B. Stier G. Sattler M. Macias M. J. (2005). Structure and dynamics of the human pleckstrin DEP domain: distinct molecular features of a novel DEP domain subfamily. *Proteins* 58 354–366. 10.1002/prot.20320 15573383

[B9] Consonni S. V. Maurice M. M. Bos J. L. (2014). DEP domains: structurally similar but functionally different. *Nat. Rev. Mol. Cell. Biol.* 15 357–362. 10.1038/nrm3791 24739740

[B10] Dahlem C. Barghash A. Puchas P. Haybaeck J. Kessler S. M. (2019). The insulin-like growth factor 2 mRNA binding protein IMP2/IGF2BP2 is overexpressed and correlates with poor survival in pancreatic cancer. *Int .J. Mol. Sci.* 20:3204. 10.3390/ijms20133204 31261900 PMC6651604

[B11] De Craene B. Berx G. (2013). Regulatory networks defining EMT during cancer initiation and progression. *Nat. Rev. Cancer* 13 97–110. 10.1038/nrc3447 23344542

[B12] Ding Y. Kantarci A. Badwey J. A. Hasturk H. Malabanan A. Van Dyke T.E. (2007). Phosphorylation of pleckstrin increases proinflammatory cytokine secretion by mononuclear phagocytes in diabetes mellitus. *J. Immunol.* 179 647–654. 10.4049/jimmunol.179.1.647 17579087 PMC2150995

[B13] Dongre A. Weinberg R. A. (2019). New insights into the mechanisms of epithelial-mesenchymal transition and implications for cancer. *Nat. Rev. Mol. Cell. Biol.* 20 69–84. 10.1038/s41580-018-0080-4 30459476

[B14] Dowler S. Currie R. A. Campbell D. G. Deak M. Kular G. Downes C. P. (2000). Identification of pleckstrin-homology-domain-containing proteins with novel phosphoinositide-binding specificities. *Biochem. J.* 351(Pt 1) 19–31. 10.1042/0264-6021:3510019 11001876 PMC1221362

[B15] Dustin M. L. Cooper J. A. (2000). The immunological synapse and the actin cytoskeleton: molecular hardware for T cell signaling. *Nat. Immunol.* 1 23–29. 10.1038/76877 10881170

[B16] Edlich C. Stier G. Simon B. Sattler M. Muhle-Goll C. (2005). Structure and phosphatidylinositol-(3,4)-bisphosphate binding of the C-terminal PH domain of human pleckstrin. *Structure* 13 277–286. 10.1016/j.str.2004.11.012 15698571

[B17] Feola M. Zamperone A. Moskop D. Chen H. Casu C. Lama D. (2021). Pleckstrin-2 is essential for erythropoiesis in β-thalassemic mice, reducing apoptosis and enhancing enucleation. *Commun. Biol.* 4:517. 10.1038/s42003-021-02046-9 33941818 PMC8093212

[B18] Guillaud-Bataille M. Brison O. Danglot G. Lavialle C. Raynal B. Lazar V. (2009). Two populations of double minute chromosomes harbor distinct amplicons, the MYC locus at 8q24.2 and a 0.43-Mb region at 14q24.1, in the SW613-S human carcinoma cell line. *Cytogenet. Genome Res.* 124 1–11. 10.1159/000200082 19372663

[B19] Hamaguchi N. Ihara S. Ohdaira T. Nagano H. Iwamatsu A. Tachikawa H. (2007). Pleckstrin-2 selectively interacts with phosphatidylinositol 3-kinase lipid products and regulates actin organization and cell spreading. *Biochem. Biophys. Res. Commun.* 361 270–275. 10.1016/j.bbrc.2007.06.132 17658464

[B20] Hu M. H. Bauman E. M. Roll R. L. Yeilding N. Abrams C. S. (1999). Pleckstrin 2, a widely expressed paralog of pleckstrin involved in actin rearrangement. *J. Biol. Chem.* 274 21515–21518. 10.1074/jbc.274.31.21515 10419454

[B21] Inazu T. Yamada K. Miyamoto K. (1999). Cloning and expression of pleckstrin 2, a novel member of the pleckstrin family. *Biochem. Biophys. Res. Commun.* 265 87–93. 10.1006/bbrc.1999.1461 10548495

[B22] Jackson S. G. Zhang Y. Bao X. Zhang K. Summerfield R. Haslam R. J. (2006). Structure of the carboxy-terminal PH domain of pleckstrin at 2.1 Angstroms. *Acta Crystallogr. D Biol. Crystallogr.* 62(Pt 3) 324–330. 10.1107/S0907444905043179 16510979

[B23] Jackson S. G. Zhang Y. Haslam R. J. Junop M. S. (2007). Structural analysis of the carboxy terminal PH domain of pleckstrin bound to D-myo-inositol 1,2,3,5,6-pentakisphosphate. *BMC Struct. Biol.* 7:80. 10.1186/1472-6807-7-80 18034889 PMC2200656

[B24] Jaramillo-Martinez V. Urbatsch I. L. Ganapathy V. (2021). Functional distinction between human and mouse sodium-coupled citrate transporters and its biologic significance: an attempt for structural basis using a homology modeling approach. *Chem. Rev.* 121 5359–5377. 10.1021/acs.chemrev.0c00529 33040525

[B25] Ji P. (2015). New insights into the mechanisms of mammalian erythroid chromatin condensation and enucleation. *Int. Rev. Cell. Mol. Biol.* 316 159–182. 10.1016/bs.ircmb.2015.01.006 25805124

[B26] Ji P. Jayapal S. R. Lodish H. F. (2008). Enucleation of cultured mouse fetal erythroblasts requires Rac GTPases and mDia2. *Nat. Cell Biol.* 10 314–321. 10.1038/ncb1693 18264091

[B27] Ji P. Murata-Hori M. Lodish H. F. (2011). Formation of mammalian erythrocytes: chromatin condensation and enucleation. *Trends Cell Biol.* 21 409–415. 10.1016/j.tcb.2011.04.003 21592797 PMC3134284

[B28] Jiang H. Xu S. Chen C. (2020). A ten-gene signature-based risk assessment model predicts the prognosis of lung adenocarcinoma. *BMC Cancer* 20:782. 10.1186/s12885-020-07235-z 32819300 PMC7439715

[B29] Kienzle N. Cross S. Young D. B. Misko I. Sculley T. B. Abrams C. S. (1997). Evidence that the expression and phosphorylation status of pleckstrin is modulated by Epstein-Barr virus in human B lymphocytes. *Blood* 89 3488–3490. 10.1182/blood.v89.9.3488 9129059

[B30] Lemmon M. A. Ferguson K. M. Schlessinger J. (1996). PH domains: diverse sequences with a common fold recruit signaling molecules to the cell surface. *Cell* 85 621–624. 10.1016/s0092-8674(00)81022-3 8646770

[B31] Lian L. Wang Y. Flick M. Choi J. Scott E. W. Degen J. (2009). Loss of pleckstrin defines a novel pathway for PKC-mediated exocytosis. *Blood* 113 3577–3584. 10.1182/blood-2008-09-178913 19190246 PMC2668855

[B32] Liu Y. Yang S. Wang F. Zhou Z. Xu W. Xie J. (2021). PLEK2 promotes osteosarcoma tumorigenesis and metastasis by activating the PI3K/AKT signaling pathway. *Oncol. Lett.* 22:534. 10.3892/ol.2021.12795 34084215 PMC8161470

[B33] Lundmark A. Davanian H. Båge T. Johannsen G. Koro C. Lundeberg J. (2015). Transcriptome analysis reveals mucin 4 to be highly associated with periodontitis and identifies pleckstrin as a link to systemic diseases. *Sci. Rep.* 5:18475. 10.1038/srep18475 26686060 PMC4685297

[B34] Luo Y. Robinson S. Fujita J. Siconolfi L. Magidson J. Edwards C. K. (2011). Transcriptome profiling of whole blood cells identifies PLEK2 and C1QB in human melanoma. *PLoS One* 6:e20971. 10.1371/journal.pone.0020971 21698244 PMC3115966

[B35] Ma A. D. Abrams C. S. (1999). Pleckstrin induces cytoskeletal reorganization via a Rac-dependent pathway. *J. Biol. Chem.* 274 28730–28735. 10.1074/jbc.274.40.28730 10497244

[B36] Ma A. D. Brass L. F. Abrams C. S. (1997). Pleckstrin associates with plasma membranes and induces the formation of membrane projections: requirements for phosphorylation and the NH2-terminal PH domain. *J. Cell. Biol.* 136 1071–1079. 10.1083/jcb.136.5.1071 9060471 PMC2132483

[B37] Mei Y. Liu Y. Ji P. (2020). Understanding terminal erythropoiesis: an update on chromatin condensation, enucleation, and reticulocyte maturation. *Blood Rev.* 46:100740. 10.1016/j.blre.2020.100740 32798012

[B38] Naume B. Zhao X. Synnestvedt M. Borgen E. Russnes H. G. Lingjaerde O. C. (2007). Presence of bone marrow micrometastasis is associated with different recurrence risk within molecular subtypes of breast cancer. *Mol. Oncol.* 1 160–171. 10.1016/j.molonc.2007.03.004 19383292 PMC5543886

[B39] Roll R. L. Bauman E. M. Bennett J. S. Abrams C. S. (2000). Phosphorylated pleckstrin induces cell spreading via an integrin-dependent pathway. *J. Cell Biol.* 150 1461–1466. 10.1083/jcb.150.6.1461 10995449 PMC2150702

[B40] Shen H. He M. Lin R. Zhan M. Xu S. Huang X. (2019). PLEK2 promotes gallbladder cancer invasion and metastasis through EGFR/CCL2 pathway. *J. Exp. Clin. Cancer Res.* 38:247. 10.1186/s13046-019-1250-8 31182136 PMC6558801

[B41] Song L. Yao J. He Z. Xu B. (2015). Genes related to inflammation and bone loss process in periodontitis suggested by bioinformatics methods. *BMC Oral Health* 15:105. 10.1186/s12903-015-0086-7 26334995 PMC4559289

[B42] Vyas V. K. Ukawala R. D. Ghate M. Chintha C. (2012). Homology modeling a fast tool for drug discovery: current perspectives. *Indian J. Pharm. Sci.* 74 1–17. 10.4103/0250-474X.102537 23204616 PMC3507339

[B43] Wang F. Zhang C. Cheng H. Liu C. Lu Z. Zheng S. (2021). TGF-beta-induced PLEK2 promotes metastasis and chemoresistance in oesophageal squamous cell carcinoma by regulating LCN2. *Cell Death Dis.* 12:901. 10.1038/s41419-021-04155-z 34601488 PMC8487427

[B44] Wang J. He Z. G. Sun B. Huang W. H. Xiang J. B. Chen Z. Y. (2021a). Pleckstrin-2 as a Prognostic Factor and Mediator of Gastric Cancer Progression. *Gastroenterol. Res. Pract.* 2021:e5527387. 10.1155/2021/5527387 34394345 PMC8360755

[B45] Wang J. Sun Z. Wang J. Tian Q. Huang R. Wang H. (2021b). Expression and prognostic potential of PLEK2 in head and neck squamous cell carcinoma based on bioinformatics analysis. *Cancer Med.* 10 6515–6533. 10.1002/cam4.4163 34331382 PMC8446404

[B46] Wang Y. Lin J. Yan K. Wang J. (2020). Identification of a robust five-gene risk model in prostate cancer: a robust likelihood-based survival analysis. *Int. J. Genomics* 2020:1097602. 10.1155/2020/1097602 32566639 PMC7285394

[B47] Wu D. M. Deng S. H. Zhou J. Han R. Liu T. Zhang T. (2020). PLEK2 mediates metastasis and vascular invasion via the ubiquitin-dependent degradation of SHIP2 in non-small cell lung cancer. *Int. J. Cancer* 146 2563–2575. 10.1002/ijc.32675 31498891

[B48] Yang Q. Li K. Li X. Liu J. (2020). Identification of key genes and pathways in myeloma side population cells by bioinformatics analysis. *Int. J. Med. Sci.* 17 2063–2076. 10.7150/ijms.48244 32922167 PMC7484674

[B49] Yang X. L. Ma Y. S. Liu Y. S. Jiang X. H. Ding H. Shi Y. (2021). microRNA-873 inhibits self-renewal and proliferation of pancreatic cancer stem cells through pleckstrin-2-dependent PI3K/AKT pathway. *Cell Signal.* 84:110025. 10.1016/j.cellsig.2021.110025 33915247

[B50] Yilmaz M. Christofori G. (2009). EMT, the cytoskeleton, and cancer cell invasion. *Cancer Metastasis Rev.* 28 15–33. 10.1007/s10555-008-9169-0 19169796

[B51] Yin H. Wang Y. Chen W. Zhong S. Liu Z. Zhao J. (2016). Drug-resistant CXCR4-positive cells have the molecular characteristics of EMT in NSCLC. *Gene* 594 23–29. 10.1016/j.gene.2016.08.043 27581786

[B52] Zhang W. Li T. Hu B. Li H. (2020). PLEK2 Gene upregulation might independently predict shorter progression-free survival in lung adenocarcinoma. *Technol. Cancer Res. Treat.* 19:1533033820957030. 10.1177/1533033820957030 33084541 PMC7588770

[B53] Zhao B. Keerthivasan G. Mei Y. Yang J. McElherne J. Wong P. (2014). Targeted shRNA screening identified critical roles of pleckstrin-2 in erythropoiesis. *Haematologica* 99 1157–1167. 10.3324/haematol.2014.105809 24747950 PMC4077076

[B54] Zhao B. Mei Y. Cao L. Zhang J. Sumagin R. Yang J. (2018). Loss of pleckstrin-2 reverts lethality and vascular occlusions in JAK2V617F-positive myeloproliferative neoplasms. *J. Clin. Invest.* 128 125–140. 10.1172/JCI94518 29202466 PMC5749534

[B55] Zhao B. Mei Y. Yang J. Ji P. (2016). Erythropoietin-regulated oxidative stress negatively affects enucleation during terminal erythropoiesis. *Exp. Hematol.* 44 975–981. 10.1016/j.exphem.2016.06.249 27364565 PMC5035599

